# Rational engineering of photosynthetic electron flux enhances light-powered cytochrome P450 activity

**DOI:** 10.1093/synbio/ysy009

**Published:** 2018-06-22

**Authors:** Adokiye Berepiki, John R Gittins, C Mark Moore, Thomas S Bibby

**Affiliations:** 1 Ocean and Earth Science, University of Southampton, Waterfront Campus, National Oceanography Centre, Southampton SO14 3ZH, UK; 2 Manchester Institute of Biotechnology, University of Manchester, Princess St, Manchester M1 7DN, UK

**Keywords:** ATP, cyanobacteria, cyclic electron flow, cytochrome P450, photosynthesis

## Abstract

In this study, we exploited a modified photosynthetic electron transfer chain (PET) in the model cyanobacterium *Synechococcus* PCC 7002, where electrons derived from water-splitting are used to power reactions catalyzed by a heterologous cytochrome P450 (CYP1A1). A simple *in vivo* fluorescent assay for CYP1A1 activity was employed to determine the impact of rationally engineering of photosynthetic electron flow. This showed that knocking out a subunit of the type I NADH dehydrogenase complex (NDH-1), suggested to be involved in cyclic photosynthetic electron flow (Δ*ndhD2*), can double the activity of CYP1A1, with a concomitant increase in the flux of electrons from photosynthesis. This also resulted in an increase in cellular adenosine triphosphate (ATP) and the ATP/nicotinamide adenine dinucleotide phosphate (NADPH) ratio, suggesting that expression of a heterologous electron sink in photosynthetic organisms can be used to modify the bioenergetic landscape of the cell. We therefore demonstrate that CYP1A1 is limited by electron supply and that photosynthesis can be re-engineered to increase heterologous P450 activity for the production of high-value bioproducts. The increase in cellular ATP achieved could be harnessed to support metabolically demanding heterologous processes. Furthermore, this experimental system could provide valuable insights into the mechanisms of photosynthesis.

## 1. Introduction

Cyanobacteria are photosynthetic bacteria of immense ecological importance but their biotechnological potential remains largely untapped. Due to their unique physiology and metabolism, cyanobacteria and other photosynthetic microbes may be particularly suitable production hosts for heterologous biosynthetic pathways (1). The promise of metabolic engineering implicitly depends on the successful expression of complex multi-enzyme pathways. In this scenario, the productivity of a pathway is limited by the enzyme with the lowest activity or expression. Given the preponderance of cytochrome P450-mediated metabolic pathways of commercial value, and recent successes in using photosynthesis to power such enzymes (2,3), the improvement of heterologous cytochrome P450 (henceforth P450) activity may be of crucial importance for realization of the biotechnological potential of cyanobacteria.

P450s are a large and diverse class of monooxygenases that play a major role in the biosynthesis and detoxification of a vast range of compounds. Biotechnological processes involving P450s have resulted in notable commercial applications (4–8). However, in their natural hosts, these enzymes are generally expressed at low levels and their heterologous expression is problematic, particularly in bacteria that lack the internal membranes required for P450 activity. Furthermore, the electron transfer required to drive P450s is often rate-limiting in common industrial hosts because nicotinamide adenine dinucleotide phosphate (NADPH) regeneration is insufficient to provide reducing equivalents to support high levels of activity (9). In addition, since P450-mediated reactions are O_2_-dependent, the microanoxic conditions that occur during heterotrophic growth in industrial bioreactors can impede product formation. Therefore, modification of the P450, the host and/or the provision of additional substrates may be required to obtain detectable activity (10). This has been a major bottleneck in attempts at metabolic engineering that depend on high-level P450 activity (1,11–14).

To overcome these issues, cyanobacteria have been utilized as hosts for P450 expression due to (i) the ample supply of photosynthetic reductant, (ii) the presence of internal thylakoid membranes as a platform for membrane protein expression and (iii) photosynthetic generation of O_2_ required for P450 catalysis (1). Moreover, it has been shown that electrons derived from photosynthesis can directly provide reducing equivalents for heterologous P450s (2,3,15–21). In these experiments, electrons from photosystem I (PSI) were coupled via ferredoxin to power heterologous P450s in a light-dependent manner. Our recent study showed that *in vivo* a heterologous P450 increased the maximum rate of electron transport from photosystem II (PSII) by >30% (3). These approaches have utilized the inherent overcapacity of the light reactions of photosynthesis, which can often produce reductant in excess of the requirements of the dark reactions and cellular metabolism. Thus, the limitations of the carbon-fixing enzyme RuBisCo, which can restrict the overall potential photosynthetic efficiency (22–25), can effectively be sidestepped and electrons that are normally ‘wasted’ can be harnessed to power useful chemical reactions (3).

Cyanobacteria and other photosynthetic organisms have evolved a number of alternate photosynthetic electron transfer (AET) pathways to mitigate the overcapacity of the light reactions of photosynthesis and prevent over-reduction of the photosynthetic electron transport chain that can lead to the generation of reactive oxygen species and photoinhibition (26). These include various water–water cycles and the cycling of electrons around PSI [cyclic electron transport (CET)], both of which result in the light-dependent formation of adenosine triphosphate (ATP) without the generation of reductant NADPH.

We reasoned that rational engineering of the photosynthetic electron flow to remove AET pathways should result in increased electron flux to a heterologous P450. To test this hypothesis, we utilized our established model system based on a *Synechococcus* PCC 7002 (henceforth *Synechococcus*) strain expressing the P450 CYP1A1, which has been engineered so that its activity is light-dependent, scaling as a saturating function of irradiance (3). CYP1A1 is a well-studied mono-oxygenase that plays an important role in the biotransformation of many drugs and toxins, and can degrade the herbicide and environmental pollutant atrazine (27). Importantly, the *in vivo* activity of CYP1A1 can be readily evaluated using a fluorescent assay (28). To increase CYP1A1 activity and to validate this approach for improving the catalytic performance of P450s in photosynthetic hosts, we focused on AET mediated by the type 1 NADPH-dehydrogenase complex (NDH-1). NDH-1 is part of the NADPH-quinone oxidoreductase family, which includes bacterial type I NADH dehydrogenase and mitochondrial complex I. Several forms of NDH-1 exist in cyanobacteria, each of which is thought to participate in distinct cellular functions including respiration, CET around PSI and CO_2_ uptake (29–31). NDH-1 oxidizes ferredoxin and returns electrons to the plastoquinone (PQ) pool, and thus constitutes a competing pathway for P450s powered by photosynthetic reductant (2,29). The M55 mutant, completely lacking a functional NDH-1 complex, shows enhanced photoevolution of hydrogen due to a reduction in O_2_ evolution and increase in the availability of reducing equivalents but displays gross defects in photosynthesis (32). Therefore, we chose to remove NdhD2, a NDH-1 subunit that would abrogate AET pathways but leave a functioning NDH-1 complex. In cyanobacteria, the NdhD2 subunit of NDH-1 is thought to function specifically in CET (29,30,33,34) and possibly in respiration (35). Furthermore, the observation that levels of *ndhD2* mRNA are increased 15-fold following high light treatment supports the notion that NdhD2 may function in CET (36). In this study, we investigate the impact of rational engineering of PET on the activity of heterologous P450 and demonstrate that significant gains can be made toward increasing the activity of this important class of enzymes.

## 2. Materials and methods

### 2.1 Chemicals and reagents

Water for the preparation of media and reagents was obtained from a Milli-Q system (Millipore). Chemicals were obtained from Sigma-Aldrich, Invitrogen or Fisher Scientific. Zeocin was purchased from Melford Biolabs. Genomic DNA was extracted with a Promega Wizard^®^ Genomic DNA Purification Kit. Polymerase chain reaction (PCR) products were purified using DNA clean and concentrator kits from Zymo Research. Oligonucleotides were purchased from Integrated DNA Technologies.

### 2.2 Culture conditions

*Synechococcus* strains were grown in A^+^ medium (37) containing sodium nitrate (1 g/l), supplemented with zeocin at 100 µg/ml where appropriate. Plates of solid A^+^ media were made following the addition of 1% agarose and 1 mM sodium thiosulphate. Cultures were grown photoautotrophically in 40 ml of liquid medium in 250 ml baffled flasks under continuous white LED illumination in an Algaetron growth chamber (PSI Instruments) at 200 µmol photons m^−2^ s^−1^ at 37°C with shaking at 200 rpm. Transformation plates were incubated at lower irradiances in a Multitron incubator (Infors) with continuous cool white fluorescent illumination at 50–70 µmol photons m^−2^ s^−1^ at 30°C. Light irradiance was measured using a Li-Cor Li-250A light sensor equipped with a Li-190SA quantum sensor. To generate growth profiles for the various strains, cells were cultured in 25 cm^2^ vented Corning tissue-culture flasks and the cell density was determined every 24 h for 7 days by measuring the OD_740nm_ using a Fluostar Optima microplate reader (BMG Labtech).

### 2.3 Strain construction

PCR primers used for cloning and genotyping are listed in Supplementary Table S1. DNA fragments representing regions of the *Synechococcus* chromosome flanking the *ndhD2* gene were generated by PCR using *Synechococcus* genomic DNA as template, Q5 High-Fidelity DNA Polymerase (NEB) and oligonucleotide primer pairs ndhD2_U_a/ndhD2_U_b (upstream fragment, 676 bp) and ndhD2_D_a/ndhD2_D_b (downstream fragment, 684 bp). In a parallel PCR using the same reaction composition, a zeocin resistance gene cassette was amplified using plasmid p75 (38) as template and the primers ndhD2_Zeo_a/ndhD2_Zeo_b (Zeo^R^, 583 bp). The three gel-purified fragments were then spliced by overlap extension (39) (SOE) using Q5 DNA Polymerase and primers ndhD2_U_a/ndhD2_D_b. *Synechococcus* strain Sy21 (3) was transformed by adding the gel-purified composite DNA fragment (1903 bp, ∼1 µg) to 3 ml of exponential phase culture (OD_730nm_ 0.6–0.7). After 16–18 h under standard growth conditions, cells were plated out on solid medium containing zeocin. Single colonies from the transformation plates were serially sub-cultured in liquid medium containing zeocin to obtain fully segregated strains. Replacement of the *ndhD2* gene with the Zeo^R^ cassette by homologous recombination and complete segregation of this knockout mutation were verified by PCR using primers ndhD2_U_a/ndhD2_D_b [wild-type (WT)—3053 bp; mutant—1903 bp].

### 2.4 Immunoblotting and quantification of CYP1A1

Whole cell extracts of *Synechococcus* strains were prepared from 40 ml cultures in log phase (OD_730nm_ 0.6–1.0). Sample extraction, SDS-PAGE, SYPRO Ruby staining and immunoblotting were carried out as described previously (3) except that a C-DiGit blot scanner was used to image immunoblots and immunoreactive bands were quantified using ImageJ software (National Institutes of Health). The difference in band intensity of CYP1A1 between strains is the average of three independent immunoblots.

### 2.5 Ethoxyresorufin O-deethylation assay for CYP1A1 activity

**Figure 1.**
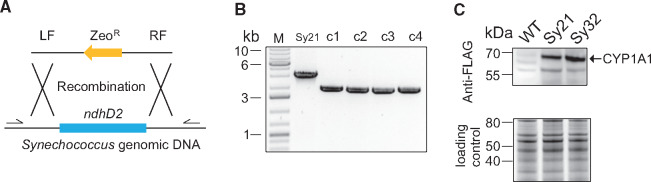
Inactivation of the *ndhD2* gene in *Synechococcus* expressing CYP1A1. (**A**) Diagram showing the *ndhD2* deletion cassette used to transform strain Sy21. Primer binding sites for genotyping are shown as arrows upstream and downstream of the *ndhD2* gene. (**B**) Genotyping of Sy21 and transformed *Synechococcus* clones by colony PCR using primers ndhD2_U_a/ndhD2_D_b. The band sizes match the predicted sizes of 3053 bp for the parent Sy21 and 1903 bp for transformants. (**C**) Expression of CYP1A1 in *Synechococcus* strains examined by immunoblotting. Shown is a representative image of an immunoblot of Sy21 versus Sy32. This experiment was repeated three times, and typical results are presented. The difference in CYP1A1 band intensity of Sy32 to Sy21 was determined in three independent immunoblots and gave an average increase in signal of 3.4%. Total protein (25 µg) from each strain was separated by SDS-PAGE and the blot probed with anti-FLAG antibody. The CYP1A1 band is indicated at the predicted mass of 61 kDa. The lower panel shows a SYPRO ruby red stained duplicate protein gel to demonstrate equal loading.

CYP1A1 activity was measured using an ethoxyresorufin O-deethylation (EROD) assay (28,40,41), as described previously (3). The end-point EROD assay was conducted over a period of incubation where EROD activity is linear (28). Cells were normalized by optical density although we show that cells have similar levels of P450 under our experimental conditions (Figure 1). Dark-/3-(3,4-dichlorophenyl)-1,1-dimethylurea (DCMU)-inhibited experiments were conducted on cells following a 1 h incubation period.

### 2.6 Quantification of ATP and NADPH

ATP levels were measured using a BacTiter-Glo^TM^ assay (Promega) based on the ATP-dependent production of luminescence. Exponentially growing cultures (OD_730_ 0.6–1.0) were adjusted to an OD_730_ of 1.0 with A^+^ medium and 100 µl aliquots of each suspension (10^7^ cells) were dispensed in triplicate into wells of a white round bottom 96-well microplate (Corning). The ATP assay was initiated by mixing 100 µl of BacTiter-Glo™ reagent with the cell suspensions and luminescence was measured using a Fluostar Optima microplate reader after incubation for 1 h in the dark at 37°C. To convert luminescence units to ATP concentration, a standard curve was generated using pure ATP (Abcam).

NADPH levels were measured using a NADP/NADPH-Glo^TM^ assay (Promega) based on the NADPH-dependent generation of luciferin, which is then quantified using luciferase, with the light signal generated being proportional to the concentration of NADP+ and NADPH in the sample. Cells from exponentially growing cultures were harvested by centrifugation at 3500 g for 10 min at room temperature (RT; 21°C). The cells were washed once in phosphate-buffered saline (PBS) then pelleted again. The pellets were resuspended in PBS to give an OD_730nm_ of 4, then 25 µl aliquots of the suspensions (10^7^ cells) were dispensed in triplicate into wells of a white round bottom 96-well microplate (Corning). The samples were processed *in situ* by the addition of 25 µl of 0.2 M NaOH containing 1% dodecyltrimethylammonium bromide followed by brief mixing on a microplate shaker to ensure homogeneity and cause cell lysis. The microplate was then lidded and incubated at 60°C for 15 min to fully lyse the cells. Following a 10 min equilibration period at RT, 50 µl of Tris/HCl solution (0.5 M Tris, 0.4 M HCl) were added to each well to neutralize the mixtures. The NADPH assay was initiated by mixing 100 µl of NADP/NADPH-Glo™ detection reagent with the samples, and following 1 h incubation in the dark at RT, luminescence was measured using a Fluostar Optima microplate reader. To convert luminescence units to NADPH concentration, a standard curve was generated using pure NADPH (Abcam). Standards were prepared in PBS and processed similarly to the experimental samples. We note that a lysis step is necessary in the quantification of ATP and NADPH from cells during which the concentrations of these metabolites may vary; however, every effort is made to ensure consistency in preparation of samples from all strains/conditions, thus ensuring comparability.

### 2.7 Biophysical measurements

Biophysical parameters were measured by kinetic fluorescence and absorbance changes (Δ*A*_830_) using samples taken from exponentially growing cultures (OD_730nm_ 0.6–0.8) after dark adaptation for 15 min. PSII kinetics were measured using the FRRf technique (42) with a FastOcean sensor integrated with an Act2 Laboratory system (Chelsea Technologies Group Ltd.), with the data used to derive PSII electron transport rates (ETR) as described previously (3). Such estimates can be subject to significant errors in the presence of substantive so-called background fluorescence (43,44). However, given the relatively small changes observed in apparent photochemical energy conversion efficiencies between strains (see below), any estimated corresponding errors in ETR were <5%. The rate of electron transport from PSI was determined by measuring redox kinetics of P700, the PSI primary donor, by following changes in absorbance at 830 nm relative to 870 nm using a Dual-PAM 100 (Heinz Walz) (45). Cells were pelleted by centrifugation at 3500 g for 10 min at RT then resuspended in A+ medium to an OD_730nm_ of 3–5 and transferred to a quartz cuvette. Fluorescence produced by an imposed actinic light gradient [fluorescence light curves (FLCs)] was measured to quantify the relative ETR from PSII (ETR(II)), while changes in Δ*A*_830_ kinetics over a similar light gradient were used to derive the relative PSI electron transport (rETR(PSI)) (45). We note that differences in reported light intensities which elicited significant responses in rates of PET and EROD assayed activity (Section 2.5) may have resulted from differences in the spectral characteristics of the different light sources and/or the complexities of establishing absolute adsorbed light doses within the complex directional light environments represented by a given apparatus. However, we further note that all direct comparisons between cell lines of either electron transport or EROD activity were performed in the same apparatus and hence all relative changes will be robust.

## 3. Results

### 3.1 Deletion of the *ndhD2* gene in *Synechococcus* expressing CYP1A1

*Synechococcus* PCC 7002 harbors a gene (SYNPCC7002_A1973) annotated as a putative NADH dehydrogenase subunit D2, which displays 69% identity to the *ndhD2* gene of *Synechocystis* PCC 6803 (30). To inactivate the *Synechococcus ndhD2* gene, we replaced the entire *ndhD2* ORF with a deletion cassette consisting of the *Streptoalloteichus hindustanus* zeocin/bleomycin resistance gene (Zeo^R^) flanked on the left (LF) by the region upstream of the target gene and on the right (RF) by the downstream region (Figure 1A). This DNA fragment was introduced into cells of *Synechococcus* strain Sy21 [a previously made strain expressing the cytochrome P450, CYP1A1 (3)] by natural transformation with selection on solid medium containing zeocin. Transformants were serially subcultured in liquid medium supplemented with zeocin to allow complete chromosomal segregation. A few resistant clones were then characterized by colony PCR to confirm the presence of the Zeo^R^ cassette and show that they were homozygous for this transgene (Figure 1B). Two clones were analyzed initially for preliminary trails and gave similar results. One of these clones was selected for further analysis and designated Sy32.

Immunoblot analysis of protein extracts prepared from strains Sy21 and Sy32 with an anti-FLAG antibody showed that the intensity of CYP1A1 was increased only slightly (<10%) by the deletion of *ndhD2* (Figure 1C). Determination of the growth rates of the WT and engineered strains showed no significant differences (Supplementary Table S2).

### 3.2 NdhD2 inactivation increases light-powered CYP1A1 activity

To determine whether the removal of alternative/competing electron dissipation pathways mediated by NdhD2 affected light-powered CYP1A1 activity, we measured the activity of this P450 in intact WT, Sy21 and Sy32 cells. This was done using the rapid and sensitive EROD assay in which ethoxyresorufin is degraded to form the fluorescent compound resorufin (28). A unique strength of this experimental system is the ability to monitor light-powered P450 activity *in vivo* as the cells are illuminated. This permits the establishment of a direct relationship between light and CYP1A1 activity (3), as photosynthetic reductant is constantly being produced and used to drive the P450 activity.

**Figure 2.**
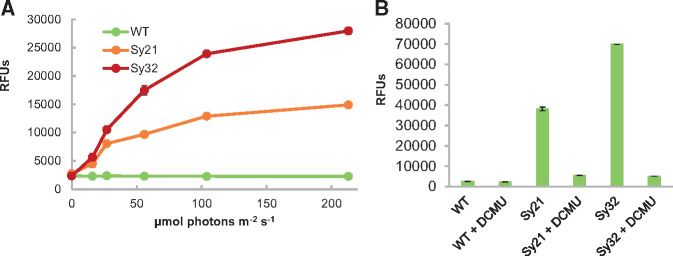
Inactivation of the *ndhD2* gene increases the activity of CYP1A1. The ethoxyresorufin O-deethylation (EROD) assay was used to measure CYP1A1 activity in live cells. CYP1A1 converts the non-fluorescent substrate ethoxyresorufin into the fluorescent product resorufin. Fluorescence measurements were made for three biological replicates 1 h after the addition of 5 μM ethoxyresorufin. Each experiment was repeated a minimum of three times and typical results are shown. Error bars represent the standard error of triplicate measurements. Values on the Y-axis are relative fluorescent units (RFUs). (**A**) CYP1A1 activity in *Synechococcus* strains at different light irradiances. Cells were dark-adapted for 1 h to deplete reducing equivalents then illuminated at different irradiances. CYP1A1 activity scales with irradiance and is increased in the absence of NdhD2. (**B**) CYP1A1 activity in *Synechococcus* strains upon inhibition of PSII activity. Cells were dark-adapted for 1 h to deplete reducing equivalents, then treated with the PSII inhibitor DCMU (3-(3,4-dichlorophenyl)-1,1-dimethylurea).

Using the EROD assay data, a light dose–response curve was produced for CYP1A1 activity (Figure 2A). This demonstrated that the inactivation of *ndhD2* increased CYP1A1 activity in strain Sy32 by almost 2-fold compared to its parent strain Sy21 at the maximum irradiance employed (213 µmol photons m^−2^ s^−1^). CYP1A1 activity was higher in this Δ*ndhD2* mutant at all irradiance levels, but the increase compared to the parent was greater at higher irradiances (80% to 88% at 56–213 µmol photons m^−2^ s^−1^) than in low light conditions (25% to 30% at 16–27 µmol photons m^−2^ s^−1^).

To identify the source of the extra electrons driving CYP1A1 activity, we treated cells with DCMU, a specific inhibitor of PSII. DCMU reduced CYP1A1 activity in strains Sy21 and Sy32 by 85.7% and 92.7%, respectively (Figure 2B). While the possibility cannot be completely excluded, the 1 h incubation period is likely too short to significantly alter protein content, therefore, the reduction in CYP1A1 activity on addition of DCMU indicates that the majority of the electrons powering this heterologous P450 are derived from light-driven PET from PSII.

### 3.3 NdhD2 inactivation increases the maximum ETR from PSII but not from PSI

**Figure 3.**
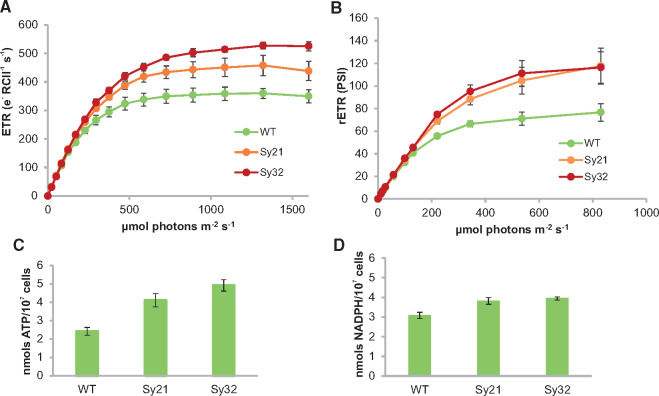
Photosynthetic physiology is altered by CYP1A1 activity and inactivation of the *ndhD2* gene. Data are the average of three independent experiments with error bars showing the standard error of triplicate measurements. (**A**) The absolute electron transport rate (ETR) from the reaction center of photosystem II (e^−^ RCII^−1^ s^−1^) for *Synechococcus* strains at different irradiances assessed by fast repetition rate fluorometry (FRRf). (**B**) The relative electron transport rate from the reaction center of photosystem I [rETR(PSI)] for *Synechococcus* strains at different irradiances assessed by Dual-PAM (pulse-amplitude modulated fluorometry). (**C**) Intracellular concentration of ATP in *Synechococcus* strains evaluated using a BacTiter-Glo^TM^ assay (Promega) based on the ATP-dependent production of luminescence. (**D**) Intracellular concentration of NADPH in *Synechococcus* strains assessed using a NADP/NADPH-Glo^TM^ assay (Promega) based on the NADPH-dependent production of luminescence.

To study the effect of *ndhD2* deletion and heterologous P450 activity on photosynthesis, we assessed relevant biophysical parameters. Fast repetition rate fluorometry (FRRf) and Δ*A*_830_ kinetic measurements were used to determine electron flux from PSII and PSI, respectively (Figure 3A and B). Compared to the WT strain, the maximum electron transport rate (max ETR) from PSII was increased by 27% in Sy21 and by 47% in Sy32 (Figure 3A). Correspondingly, the saturating light intensity (Ek) was increased by 19% for Sy21 and 40% for Sy32, indicating the enhanced capacity of these strains, particularly the latter, to process electrons from PSII (Table 1). Photosynthetic energy conversion efficiency (*F*_v_/*F*_m_) was similar in the WT (0.459) and Sy21 (0.453) strains (Table 1), but was increased in Sy32 (0.499), potentially suggestive of a more oxidized PQ pool under dark acclimated conditions in this strain, although this did not enhance its growth rate (Supplementary Table S2). To investigate PSI kinetics, we assessed the P700 redox state and relative ETR using Δ*A*_830_ measurements (45). Compared to the WT, the relative ETR (rETR) from PSI was increased by 63% and 68% in Sy21 and Sy32, respectively (Figure 3B). Therefore, CYP1A1 activity increases electron transport through PSII and PSI; deletion of *ndhD2* further increases electron flux through PSII but not PSI.

**Table 1:** *F*_v_/*F*_m_ and electron transport rates for PSII and PSI of *Synechococcus* strains

Strain	Photosynthetic energy conversion efficiency (*F*_v_/*F*_m_)	Maximum electron transport rate (e^−^ RCII^−1^ s^−1^)	Maximum relative electron transport rate (PSI)
WT	0.459 ± 0.001	353.1 ± 23.2	76.7 ± 6.01
Sy21	0.453 ± 0.007	447.0 ± 32.0	117.6 ± 15.87
Sy32	0.499 ± 0.006	518.7 ± 11.4	116.4 ± 14.01

The values shown are the means from three independent experiments ± the standard error where appropriate.

### 3.4 Light-powered P450 activity increases cellular ATP and NADPH concentrations

Given the apparent modification of photosynthetic physiology in the engineered strains, we examined the levels of the two photochemical reaction products ATP and NADPH. Previously, we hypothesized that an increase in PSII max ETR mediated by the activity of CYP1A1 should be accompanied by an increase in cellular ATP levels, since at high irradiances, electron sink limitation has been relieved by the presence of the P450, potentially oxidizing the PQ pool and allowing increased activity of PSII (3). Indeed, compared to the WT, the measured cellular ATP concentration was increased in the Sy21 and Sy32 strains by 72% and 107%, respectively (Figure 3C). Thus, the ATP concentration seems to be related to the activity of CYP1A1.

Cellular NADPH levels were also raised in the engineered strains compared to the WT, but to a lesser extent, with increases of 23% in Sy21 and 28% in Sy32 (Figure 3D). The increase in NADPH is lower than that of ATP, thus the ATP:NADPH ratios of Sy21 (1.05) and Sy32 (1.23) were significantly higher than the WT (0.76), and appear to be related to the activity of the introduced heterologous electron sink, CYP1A1.

## 4. Discussion

In this study, we attempted to identify and overcome the rate-limiting electron supply to a light-powered heterologous P450 expressed in *Synechococcus* in order to enhance its activity. CYP1A1 activity was nearly doubled by the deletion of *ndhD2* (Figure 2A). The increased CYP1A1 activity in strain Sy32 indicates that more electrons from the photosynthetic light reactions are available to power this P450 when *ndhD2* is deleted. This is consistent with the suggestion that NdhD2 is involved in CET (29,36) and that in strain Sy21 this subunit of the NADPH-dehydrogenase complex is competing with the P450 for electrons (3,18). The greater difference in P450 activity between Sy21 and Sy32 under high irradiances further supports this conclusion because AET such as CET are utilized more when processes downstream of PSI are saturated, such as under high light conditions (29,46). Taken together, these data suggest that the provision of electrons to CYP1A1 controls its activity and that more electrons are available to drive this P450 when CET is reduced. The extra electrons are not from respiration as Sy21 and Sy32 treated with the PSII inhibitor DCMU show similar levels of residual enzyme activity (Figure 2B). The similarity of this value in Sy21 and Sy32 suggests that an equivalent contribution is made by respiration in driving P450 activity in both strains.

Investigation of photosynthetic physiology in the engineered strains demonstrated that the introduction of CYP1A1 as an additional electron sink raised the photosynthetic capacity of the *Synechococcus* cells by increasing the ETR of both PSII and PSI (Figure 3A and B). However, while deletion of *ndhD2* in the presence of CYP1A1 further increased the ETR of PSII in Sy32, the ETR of PSI remained the same as in Sy21. The PSI ETR measurements in the absence of NdhD2 are thus consistent with the notion that reduced CET in strain Sy32 has been complemented by higher CYP1A1 activity (Figure 2A). As a further experiment, we also analyzed the electron flux through PSII of Sy38 [a ΔndhD2 mutant in the WT background lacking CYP1A1 (Supplementary Figure S1)]. Sy38 was observed to have a 33% higher maximum electron flux through PSII compared to the WT, which, while potentially supporting our suggestion that reduction of CET can allow increased LET from PSII [as observed in Sy32 (Figure 3B)], the comparison should be treated with caution as the physiology of an Δ*ndhD2* mutant may be very different to when the additional artificial electron sink (CYP1A1) is present. Although the majority of available evidence points to *ndhD2* being involved in CET, the subunit may have other roles (29–31). The evidence presented here, provides further indirect evidence and supports the notion that ndhD2 does have a role in CET.

It is also interesting to consider if addition of a chemical inhibitor of CET (e.g. *DBMIB*) would produce a similar result to the ΔndhD2 mutant i.e. increased P450 activity. This experiment is worthy of future study, however, an analysis of the physiology of a specific mutant with reduced CET in contrast to a short-term chemical inhibition of CET with DBMIB may prevent meaningful comparison (47).

Our data suggest that the introduction of an additional electron sink has modified the balance of photochemical products, leading to the production of more ATP (Figure 3C). These findings highlight the potential to rationally engineer photosynthesis in order to increase the activity of a specific pathway and alter the ATP:NADPH ratio, which may be beneficial for biotechnological applications of cyanobacteria that have a high ATP demand, such as fatty acid synthesis (48). Levels of NADPH were also increased (Figure 3D). This was unexpected because CYP1A1 consumes photosynthetic reductant (3). However, NADPH is not used directly by CYP1A1 and the introduction of a strong electron sink into PET may alter the regeneration of NADPH from NADP^+^. Recently, it was shown that the introduction of a NADPH sink in the form of a metabolic pathway enhanced growth and biomass formation in *Synechocystis* PCC 6803 under high irradiances and increased the ATP:NADPH ratio (49). Therefore, reengineering the balance of photochemical products may be a useful approach to alter photosynthetic processes and the performance of photosynthetic organisms.

**Figure 4.**
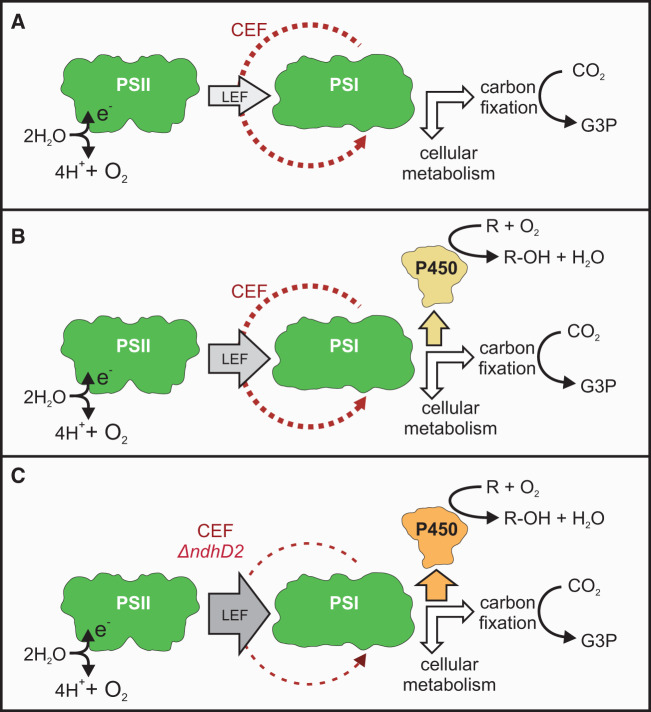
Rational engineering of photosynthesis to enhance P450 activity: model of linear and cyclic electron transport based on experimental data. (**A**) Photosynthetic electron flux in WT *Synechococcus*. (**B**) Photosynthetic electron flux with a P450 acting as an electron sink. (**C**) Photosynthetic electron flux in the absence of cyclic electron flow with a P450 acting as an electron sink. The increasing flux of electrons from PSII (from A–C) is denoted by gray arrows, electrons from cyclic electron flow are shown as red arrows (decreasing in C, dashed), the linear electron flux from PSI is indicated by white arrows, orange arrows indicate the electron flux to P450 (increasing in A–C).

Based on the outcome of the rational engineering of PET described here, we propose a simplified model of this process (Figure 4). For the purposes of this model, we assume that the ratios of PSII and PSI concentrations within the cell remain constant and that the flux of electrons in water-water cycles such as those mediated by Flv1 and Flv3 (50) remains unchanged. LET and CET work in parallel to provide ATP and NADPH to the cell (Figure 4A) (51,52). Electron transfer from PSII processes electrons from water only to LET; while electron transfer from PSI includes flux from both LET and CET. At high light, CET is increased to protect PSII from over-reduction and potential photoinhibition, and as the cell’s demand for reductant from LET is saturated (29,46).

Engineering of an additional electron sink (P450) powered by photosynthetic electrons (Figures 2A and 4B), downstream of PSI but before RuBisCo, increases the ETR through PSII and PSI to provide additional electrons to power this supplementary activity (Figures 2A and 3). Removal of NdhD2 (thought to be required for CET) (Figure 4C), eliminates a competing sink of electrons from PSI. As the P450 catalyzes a linear and light-dependent electron transport pathway that can be blocked by DCMU, the additional electrons needed to produce the observed increase in P450 activity can only be supplied by increased LET, hence the PSII ETR is higher (Figure 3). As subunits involved in CET by PSI have been removed, we propose that the existing PSI pool may now have the capacity to support the required additional LET and hence P450 activity, without a further increase in PSI ETR, as observed (Figure 3).

Such a model would also be consistent with the increases in ATP concentration observed in Sy21 and Sy32 (Figure 3C), as a larger fraction of the capacity of PSI is potentially used in LET to support CYP1A. Specifically, LET involving both PSII and PSI produces 3 ATPs per 4 electrons transferred through both complexes, while CET produces 2 ATPs per 4 electrons through PSI (53). Consequently, the increase in PSII ETR and hence LET from WT through Sy21 to Sy32 (Figure 3A), would be expected to be accompanied by increased ATP production (Figure 3C). In the case of Sy32, this extra ATP production through LET more than offsets the lower ATP production from reduced CET as overall PSI ETR appears to be repurposed from the latter to the former (Figure 4). To confirm this proposed model many additional experiments could be conducted that are beyond the scope of the current study. For example, while we demonstrate the abundance of P450 is similar between mutants (Figure 1) the arrangement of PSI and P450 and/or specific activity of P450 may have changed owing to either membrane scale changes induced by modification of the NDH-complex or for example the availability of heme. A study of the membrane level organization of photosystems and P450 in these mutants could yield important insights—both into the function of the presented mutants and that manipulation of PSI:P450 coupling could be a further method in increase flux to artificial electron sinks (17,18).

Overall, our proposed model is supported by our experimental data and fits with the current understanding of PET (Figure 4). It also highlights how this system might be used as an *in vivo* real-time tool for the rational improvement of photosynthesis. Such improvements may be achieved through manipulation of the antenna cross-section of the photosystems, the PSII:PSI ratio, or the modification of other dissipation pathways such as those mediated by Ptox and ATRO (26).

## 5. Conclusions

We have shown that the removal of natural pathways for the dissipation of reductant from the light reactions of photosynthesis is a rational means of manipulating and increasing the photosynthetic electron flow to a desired process. Our success in streamlining the photosynthetic host to improve light-driven P450 activity highlights the potential of cyanobacteria as green biofactories in biotechnology. In addition, we have demonstrated how this system, where P450 activity is analyzed *in vivo* and in real time, can provide novel insights into the mechanism of PET. Our findings represent a significant step towards the rational engineering of photosynthesis, the pivotal biochemical reaction on the planet.

## Supplementary Data

Supplementary Data are available at SYNBIO Online.

## Funding

BBSRC grant (www.bbsrc.ac.uk) [BB/M011305/1, BB/P019331/1 to T.S.B.]. A.B. conceived the project. T.S.B. supervised the project. A.B. and T.S.B. designed the experiments. A.B. performed most of the experiments. J.R.G. constructed the *ndhD2* deletion cassette. A.B., J.R.G., C.M.M., and T.S.B. analyzed and interpreted the data. A.B. and T.S.B. wrote the paper and all authors critically reviewed, edited and approved the manuscript.

*Conflict of interest statement*. None declared.

## Supplementary Material

Supplementary Data
