# Supplementary material for: Rational engineering of photosynthetic electron flux enhances light-powered cytochrome P450 activity
Source: Synth Biol (Oxf). 2018 Jun 22;3(1):ysy009. doi: 10.1093/synbio/ysy009 (PMC7445785; doi:10.1093/synbio/ysy009)
Supplement: Supplementary Data [file ysy009_supp.pdf]

Supplementary material

Table S1. Primers used to construct the *ndhD2* deletion cassette and for genotyping.

**Primer name**	**Nucleotide sequence 5'-3'**
ndhD2_U_a	GCGCCTATTTTGATGGGGAG
ndhD2_U_b	TATTATTGCGTTCGCCTCCG
ndhD2_Zeo_a	CGGAGGCGAACGCAATAATAGTGTTGACAATTAATCATCGGCA
ndhD2_Zeo_b	GCTACTTAGAGCCCATCCGTGCGTGAATGTAAGCGTGACA
ndhD2_D_a	ACGGATGGGCTCTAAGTAGC
ndhD2_D_b	CACAAATGTTTCCTGGCCCA

Table S2. Growth rates of *Synechococcus* strains.

**Strain**	**Specific growth rate μ (day^{-1})**
WT	0.77 ± 0.063
Sy21	0.78 ± 0.054
Sy32	0.75 ± 0.031

The values shown are the means from three independent experiments ± the standard error.

Supplementary Material Figure 1

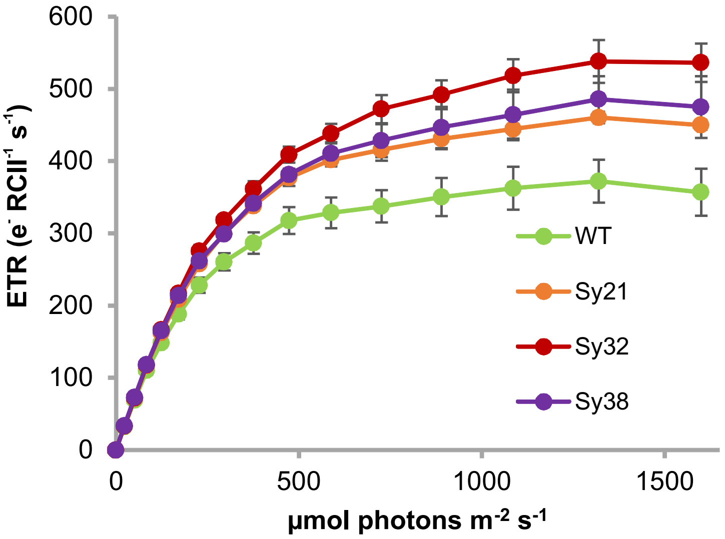

Supplementary Material Figure 1. Photosynthetic physiology is altered by the inactivation of the *ndhD2* gene. The *ndhD2* gene was deleted in WT *Synechococcus* as an additional control and the resulting strain was designated Sy38. Data are the average of three independent experiments with error bars showing the standard error of triplicate measurements. The absolute electron transport rate (ETR) from the reaction center of photosystem II (e^- $RCII^{-1}$ s^{-1}) for *Synechococcus* strains at different irradiances assessed by fast repetition rate fluorometry (FRRf).
